# Involvement of A20 in the molecular switch that activates the non-canonical NF-кB pathway

**DOI:** 10.1038/srep02568

**Published:** 2013-09-06

**Authors:** Noritaka Yamaguchi, Masaaki Oyama, Hiroko Kozuka-Hata, Jun-ichiro Inoue

**Affiliations:** 1 Division of Cellular and Molecular Biology, Department of Cancer Biology, Institute of Medical Science, University of Tokyo, Shirokanedai, Minato-ku, Tokyo, 108-8639, Japan; 2 Medical Proteomics Laboratory, Institute of Medical Science, University of Tokyo, Shirokanedai, Minato-ku, Tokyo, 108-8639, Japan; 3 Current address: Department of Molecular Cell Biology, Graduate School of Pharmaceutical Sciences, Chiba University, Chiba 260-8675, Japan.

## Abstract

The non-canonical NF-κB pathway is crucial for the immune system. A critical event in activation of the non-canonical pathway is the attenuation of NF-κB-inducing kinase (NIK) degradation, which is promoted by continuous polyubiquitination of NIK catalyzed by the NIK ubiquitin-ligase complex composed of cellular inhibitor of apoptosis protein 1 and 2 (cIAP1/2), TNF receptor-associated factor 2 (TRAF2), and TRAF3. However, the molecular mechanism of stimulation-dependent NIK stabilization remains poorly understood. Here, we show that A20, a ubiquitin-editing enzyme, promotes efficient activation of the non-canonical pathway independent of its catalytic activity. A20 directly binds to cIAP1 through the seventh zinc finger of A20, resulting in dissociation of the TRAF2/TRAF3 interaction, thereby inactivating the ligase complex to stabilize NIK. Given that A20 negatively regulates the canonical pathway, A20 is likely involved in the molecular switch that promotes the transition from canonical to non-canonical activation for proper control of the immune system.

The transcription factor nuclear factor-κB (NF-κB) controls the genes necessary for inflammation, immunity, and cell survival^1^. The NF-κB family is composed of five members, including RelA, RelB, and c-Rel and the precursor and processed products of the *NFKB1* (p105/p50) and *NFKB2* (p100/p52) genes. Although these proteins form homodimers or heterodimers in various combinations, the main complexes that activate transcription are the p50/RelA and the p52/RelB complexes, which are sequestered in the cytoplasm by the inhibitor of NF-κB (IκB) family or a p100 protein, respectively, in unstimulated cells^2^. Two distinct pathways have been proposed for NF-κB activation. The canonical pathway is activated by cytokines such as tumor necrosis factor (TNF)-α and interleukin (IL)-1 and bacterial products and plays a critical role in the expression of inflammatory cytokines and inhibition of apoptosis. Stimulation with these ligands leads to the activation of the IκB kinase (IKK) complex, which consists of the catalytic subunits IKKα and IKKβ and the regulatory subunit NF-κB essential modulator (NEMO). IKK then phosphorylates IκBs and targets them for K48 polyubiquitination, which leads to the degradation of ubiquitinated IκBs by the proteasome, allowing the p50/RelA complex to enter the nucleus and activate target genes^2^. Alternatively, the non-canonical pathway, which is required for lymphoid organogenesis, is activated by stimulation by lymphotoxin-β receptor (LTβR), CD40, receptor activator of NF-κB (RANK), fibroblast growth factor-inducible 14 (Fn14), or B cell activating factor belonging to TNF family receptor (BAFFR). This pathway involves activation of the IKKα homodimer, which then phosphorylates the C-terminal domain of p100 and targets it to proteasome-dependent processing to generate p52, allowing the p52/RelB complex to enter the nucleus and activate target genes^3^,^4^.

The activation of the IKK complex is controlled by post-translational modifications of signaling components, including phosphorylation and ubiquitination^5^. In the canonical pathway, a protein complex composed of cellular inhibitor of apoptosis protein 1 and 2 (cIAP1/2) and TNF receptor-associated factor 2 (TRAF2), together with the E2 enzyme UBC5, induces K63 polyubiquitination of receptor-interacting protein kinase 1 (RIP1) upon TNF-α stimulation^6^. The K63 polyubiquitin chain does not induce proteasomal degradation but acts as a platform for the formation of active signal complexes consisting of transforming growth factor β-activated kinase 1 (TAK1), TAK1-binding (TAB) 2/TAB3, and the IKK complex; TAB2/3 and NEMO bind to K63 polyubiquitin chains via their ubiquitin-binding domains. The formation of this complex leads to the activation of TAK1, which then phosphorylates and activates IKKβ^7^,^8^,^9^. Although the mechanism remains to be elucidated, UBC5 and cIAP1 also support conjugation of RIP1 with non-K63 polyubiquitin chains, which results in IKKβ activation^6^. In addition to TAK1 activation, IKK activation requires the stimulation-induced conjugation of linear polyubiquitin chains to NEMO catalyzed by the linear ubiquitin chain assembly complex (LUBAC), which may induce oligomer formation or a conformational change in NEMO to activate the IKK complex^10^,^11^,^12^,^13^,^14^. In the non-canonical pathway, NF-κB-inducing kinase (NIK) phosphorylates and activates IKKα homodimer, which phosphorylates p100 to promote p100 processing to p52. Under unstimulated conditions, NIK is persistently degraded by the proteasome due to K48 polyubiquitination by cIAP1/2; the TRAF2/TRAF3 heterodimer acts as a molecular bridge between NIK and cIAP1/2 (Ref. 3,4). Stimulation that activates the non-canonical pathway induces stabilization and activation of NIK, occasionally with concomitant degradation of TRAF2 and TRAF3 (Ref. 15,16). Although the molecular switch that attenuates NIK degradation is a critical component in understanding the non-canonical pathway, its mechanism remains to be elucidated.

A20 is a ubiquitin-editing enzyme that potently suppresses the canonical pathway^17^. The N-terminal ovarian tumor (OTU) domain of A20 can deubiquitinate K63 polyubiquitination of RIP1, and the C-terminal zinc-finger (ZnF) region subsequently acts as an E3 ligase to add the K48 polyubiquitin chain to RIP1, thereby promoting its proteasomal degradation^18^. A20 also inactivates the E2/E3 ubiquitination complexes required for activation of the canonical pathway by inhibiting the association of UBC13 with TRAF6, TRAF2 with cIAP1/2, or UBC5C with TRAF6, and subsequently promoting proteasomal degradation of E2 enzymes^19^. Moreover, A20 impairs IKKβ activation by binding to a K63 or linear polyubiquitin chain without requiring the deubiquitinase and ubiquitin ligase activities of A20 (Ref. 20–22). These previous studies clarified the inhibitory roles of A20 in the canonical pathways. However, the roles of A20 in the non-canonical pathway are largely unknown. In this paper, we show, for the first time, that A20 functions as a positive regulator of the non-canonical pathway by promoting the stimulation-dependent stabilization of NIK.

## Results

### A20 is crucial for the efficient activation of the non-canonical NF-κB pathway

**Figure 1:**
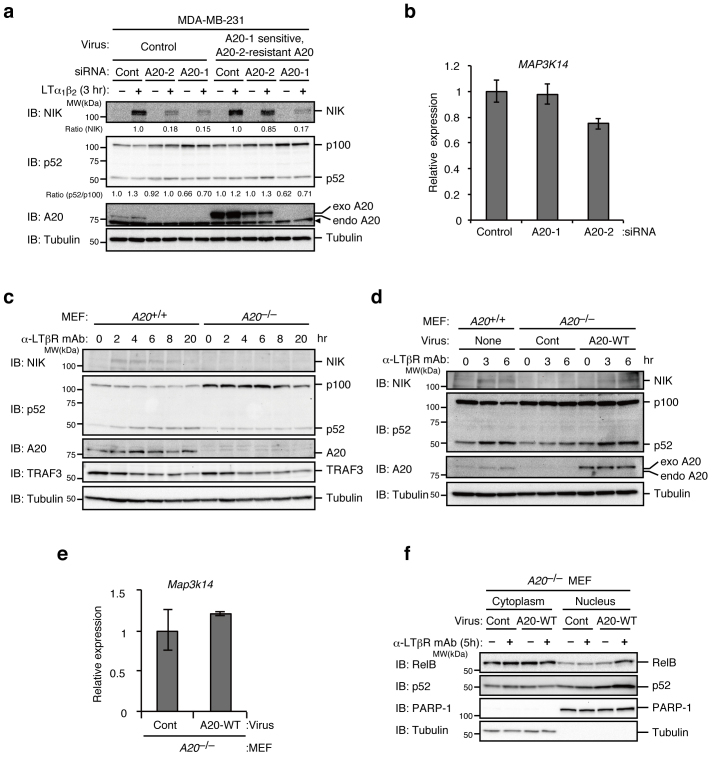
A20 is required for efficient activation of the non-canonical NF-κB pathway upon LTβR stimulation. (a) MDA-MB-231 cells infected with control virus or virus expressing siRNA A20-1-sensitive, A20-2-resistant human A20 were transfected with the indicated siRNAs. After 72 hr, the cells were untreated or treated with recombinant LTα1β2 (500 ng/ml) for 3 hr. Cell lysates were then prepared and subjected to immunoblotting with the indicated antibodies. Amounts of NIK and ratios of p52 to p100 are shown as relative values, with the corresponding values from the unstimulated control siRNA-transfected cells set to 1. (b) MDA-MB-231 cells were transfected with the indicated siRNAs. After 72 hr, total RNA was extracted and the expression levels of NIK (*MAP3K14*) mRNA were measured by real-time RT-PCR. The level of GAPDH mRNA expression was used to normalize the data. The expression level of NIK mRNA in control cells was set to 1. (c) and (d) *A20*^+/+^ MEFs and *A20*^−/−^ MEFs (c) or *A20*^+/+^ MEFs or *A20*^−/−^ MEFs infected with control virus or virus expressing mouse wild type A20 (d) were stimulated with an agonistic anti-LTβR mAb (1 μg/ml) for the indicated time. Cell lysates were prepared and subjected to immunoblotting as in (a). (e) Total RNA was extracted from *A20*^−/−^ MEFs infected with control virus or virus expressing mouse wild type A20, and real-time RT-PCR was performed as in (b), except that the β-actin mRNA expression level was used to normalize the data. The NIK mRNA expression level in *A20*^−/−^ MEFs infected with control virus was set to 1. (f) *A20*^−/−^ MEFs infected with control virus or virus expressing mouse wild type A20 were either unstimulated or stimulated with an agonistic anti-LTβR mAb (1 μg/ml) for 5 hr. Cells were then fractionated into cytoplasmic and nuclear fractions for subsequent immunoblotting as in (a). Tubulin is shown as a cytoplasmic marker, and PARP-1 is shown as a nuclear marker. The results shown in (b) and (e) indicate the mean ± SD (n = 3). The depicted results are representative of three independent experiments.

To explore novel roles of A20, we first generated human HEK293T cells expressing A20 N-terminally fused to a tandem affinity purification (TAP)–tag consisting of FLAG and double strep tags (TAP-A20-overexpressing HEK293T cells) by retrovirus-mediated gene transfer. A20-containing complexes were then isolated from the cells and subjected to mass spectrometry analysis. cIAP1 and TRAF2 are found to be components of the A20-containing complex, which led us to think that A20 might be involved in the non-canonical NF-κB pathway. To analyze the involvement of A20 in the non-canonical pathway, the human breast cancer cell line MDA-MB-231 was transfected with control small interfering RNA (siRNA) or two distinct siRNAs for A20 (A20-1 and -2) and then stimulated with recombinant LTα_1_β_2_. Both siRNAs for A20 were able to dramatically reduce the expression of A20 (Fig. 1a) and efficiently block the known function of A20; activation of the canonical pathway (phosphorylation of IKK and IκBα) by IL-1 in the late phase (120 min after stimulation) was significantly enhanced in these A20-knockdown cells (Supplementary Fig. S1)^19^. Ligation of LTβR resulted in the accumulation of NIK protein and the enhanced processing of p100 to p52 in the control siRNA-treated cells while these processes were significantly inhibited in A20-knockdown cells (Fig. 1a). NIK mRNA expression was not significantly affected by A20-1 siRNA (Fig. 1b). Although A20-2 siRNA slightly reduced (approx. 20%) NIK mRNA expression (Fig. 1b), this siRNA caused dramatic reduction (more than 80%) in NIK protein expression (Fig. 1a), indicating that the reduction in NIK mRNA accounts for little if any of the NIK protein reduction seen in the A20 knockdown cells. Therefore, we speculated that A20 regulates NIK protein expression at the translational or post-translational level. The specificity of this knockdown experiment was confirmed by a rescue experiment in which an siRNA A20-1-sensitive, A20-2-resistant A20 expression construct was introduced into MDA-MB-231 cells (Fig. 1a). Furthermore, LTβR-stimulation failed to induce NIK accumulation and efficient p100 processing in *A20*^−/−^ mouse embryo fibroblasts (MEFs), whereas their robust induction was observed in wild-type MEFs (Fig. 1c). Retrovirus-mediated introduction of A20 into *A20*^−/−^ MEFs restored the LTβR-induced NIK accumulation and p100 processing (Fig. 1d) without affecting NIK mRNA levels (Fig. 1e). LTβR-stimulation-induced nuclear translocation of p52 and RelB, major NF-κB subunits that are activated in the non-canonical pathway, was abolished in *A20*^−/−^ MEFs but was restored by reintroducing A20 (Fig. 1f). Taken together, A20 is crucial for the efficient activation of the non-canonical NF-κB pathway induced by LTβR signaling.

**Figure 2:**
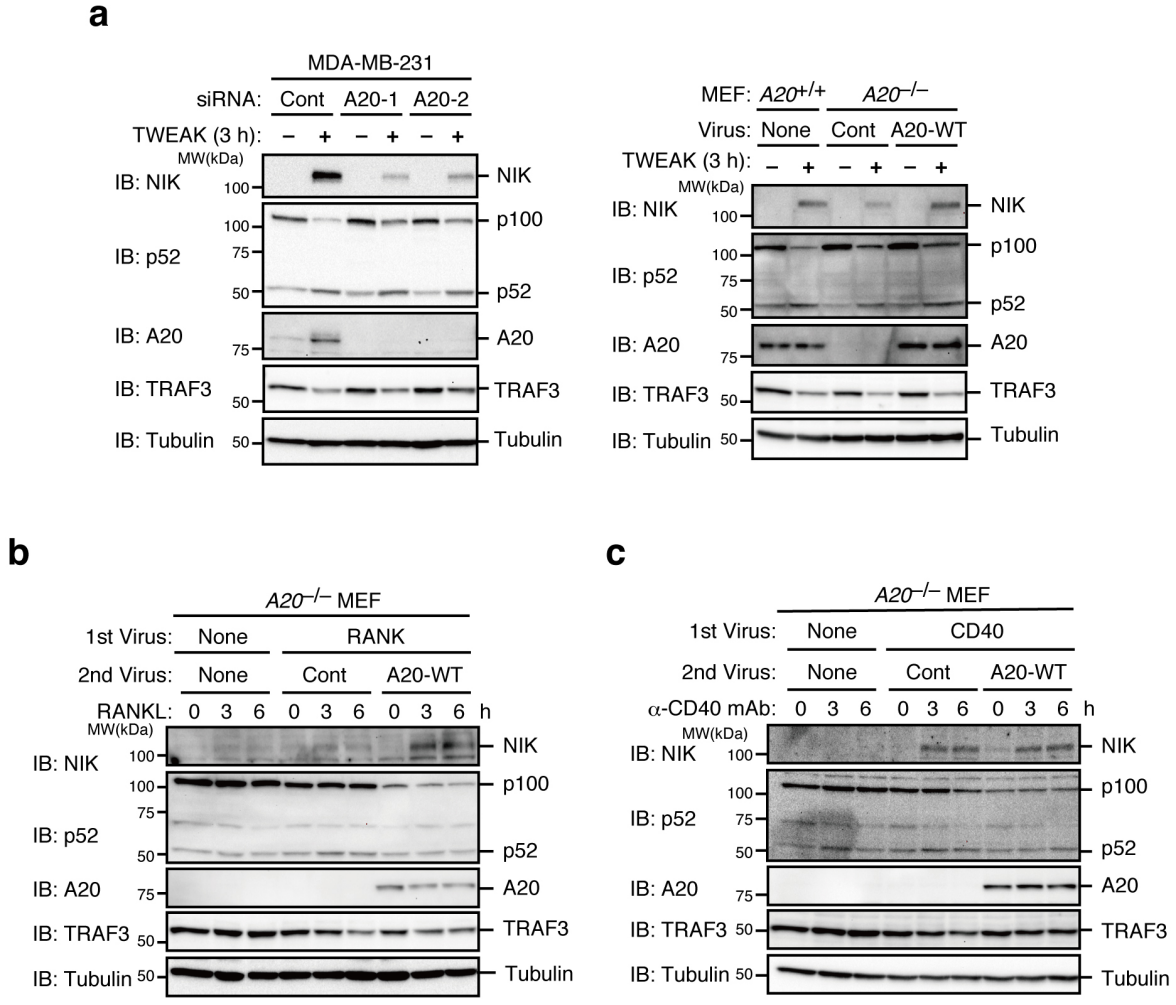
A20 is required for efficient activation of the non-canonical NF-κB pathway upon Fn14 or RANK stimulation but is dispensable for activation upon CD40 stimulation. (a) MDA-MB-231 cells were transfected with the indicated siRNAs. After 72 hr, the cells were untreated or treated with recombinant TWEAK (100 ng/ml) for 3 hr. Cell lysates were then prepared and subjected to immunoblotting (left). *A20*^+/+^ MEFs or *A20*^−/−^ MEFs infected with control virus or virus expressing wild type A20 were stimulated with recombinant TWEAK for 3 hr. Cell lysates were prepared and subjected to immunoblotting (right). (b) *A20*^−/−^ MEFs infected with virus expressing RANK were further infected with either control virus or virus expressing mouse wild type A20. Parental *A20*^−/−^ MEFs and doubly infected cells were stimulated with recombinant RANKL (100 ng/ml) for the indicated time. Cell lysates were prepared and subjected to immunoblotting. (c) *A20*^−/−^ MEFs infected with virus expressing CD40 were further infected with either control virus or virus expressing mouse wild type A20. Parental *A20*^−/−^ MEFs and doubly infected cells were stimulated with an agonistic anti-human CD40 mAb (Clone G28-5, 1 μg/ml) for the indicated time. Cell lysates were prepared and subjected to immunoblotting. The depicted results are representative of three independent experiments.

We next sought to determine whether A20 is commonly involved in the non-canonical NF-κB pathways of various members of the TNF receptor superfamily (TNFRSF). Fibroblast growth factor-inducible 14 (Fn14)-induced NIK accumulation and p100 processing in response to stimulation with tumor necrosis factor-like weak inducer of apoptosis (TWEAK) were significantly reduced in the A20-knockdown MDA-MB-231 cells and *A20*^−/−^ MEFs but were restored by their complementation with A20 (Fig. 2a). Similarly, efficient NIK accumulation and p100 processing induced by the stimulation of receptor activator of NF-κB (RANK) with RANK ligand (RANKL) depended on A20 (Fig. 2b). By contrast, CD40 stimulation similarly induced NIK accumulation in the presence or the absence of A20 (Fig. 2c). Taken together, these results strongly suggest that A20 acts as a positive regulator for most but not all of the non-canonical NF-κB pathways. CD40 may have an A20-independent regulatory mechanism for its non-canonical pathway.

### Overexpression of A20 leads to activation of the non-canonical NF-κB pathway

**Figure 3:**
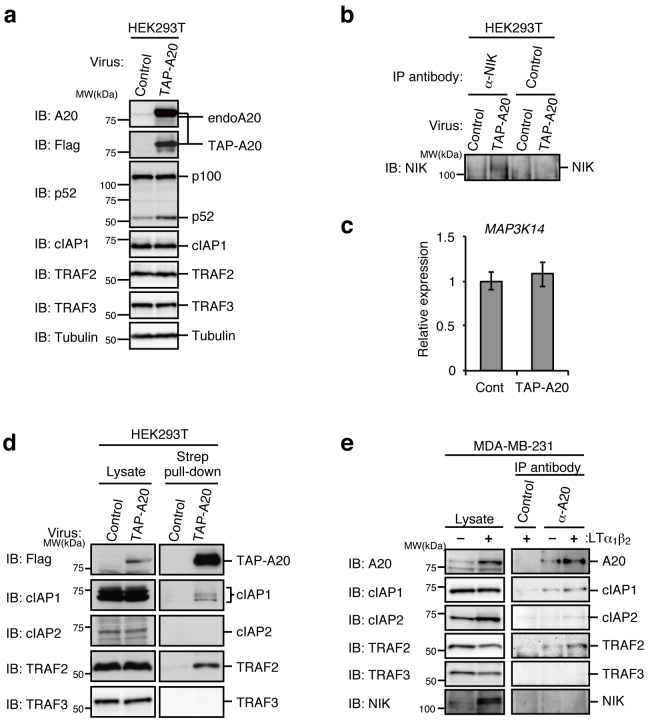
Overexpression of A20 results in activation of the non-canonical NF-κB pathway. (a) HEK293T cells infected with control virus or virus expressing TAP-tagged human A20 (TAP-A20) were lysed and subjected to immunoblotting. (b) The cell lysates prepared in (a) were subjected to immunoprecipitation with anti-NIK or anti-myc (control) antibodies. Immunoprecipitates were subjected to immunoblotting with an anti-NIK antibody. (c) Total RNA was extracted from HEK293T cells infected with control virus or virus expressing TAP-A20, and the expression levels of NIK (*MAP3K14*) mRNA were measured by real-time RT-PCR. The level of GAPDH mRNA expression was used to normalize the data. The expression level of NIK mRNA in cells infected with the control virus was set to 1. The result indicates the mean ± SD (n = 3). (d) The cell lysates prepared from HEK293T cells infected with control virus or virus expressing TAP-A20 were subjected to affinity pull-down with Strep-Tactin Superflow beads. The lysates and precipitates were subjected to immunoblotting. (e) MDA-MB-231 cells were untreated or treated with recombinant LTα1β2 (500 ng/ml) for 3 hr. Cell lysates were then prepared and immunoprecipitated with anti-A20 or anti-myc (control) antibodies. The lysates and precipitates were subjected to immunoblotting. The depicted results are representative of three independent experiments.

To elucidate the function of A20 in the non-canonical pathway, the expression levels of proteins involved in the non-canonical pathway were compared between control and TAP-A20-overexpressing HEK293T cells used for the initial analysis of the A20-containing complexes. Interestingly, A20 overexpression resulted in a significant enhancement of p100 processing (Fig. 3a) and an increase in NIK accumulation (Fig. 3b), although the mRNA level of NIK was not significantly affected (Fig. 3c). Given that NIK accumulation and p100 processing are induced in response to extracellular stimuli that activate the non-canonical pathway^3^,^4^, overexpression of A20 may trigger core molecular events required for the activation of the non-canonical pathway in the absence of extracellular stimuli. However, the degradation of TRAF3 observed in the activation of the non-canonical pathway induced by extracellular stimulation did not occur as a result of A20 overexpression (Fig. 3a), while TRAF3 degradation was similarly induced upon LTβR stimulation in the presence or absence of A20 (Fig. 1c and 2a, b). Given that stimulation-induced NIK accumulation was not observed in *A20*^−/−^ MEFs, these results suggest that ubiquitination and subsequent degradation of TRAF3 could occur without attenuation of NIK ubiquitination.

cIAP1/2 are ubiquitin ligases of NIK, whereas TRAF2/TRAF3 heterodimers act as the molecular bridge that recruits cIAP1/2 to NIK, thereby forming the cIAP1/2-TRAF2-TRAF3-NIK complex to conjugate the K48 polyubiquitin chain to NIK. This polyubiquitination results in constitutive proteasomal degradation of NIK in unstimulated cells^15^,^16^. Upon stimulation, cIAP1/2 may no longer recognize NIK as a substrate, thereby inducing NIK accumulation. Given that A20 is likely to regulate NIK protein expression translationally or post-translationally, we first checked whether A20 regulates the stability of NIK protein. MDA-MB-231 cells transfected with either A20-2 siRNA or control siRNA were treated with MG132, a proteasome inhibitor, to induce NIK accumulation. The cells were then further incubated with MG132-free media supplemented with cycloheximide, a protein synthesis inhibitor, to analyze NIK protein stability. A20 knockdown shortened the half-life of the NIK protein (Supplementary Fig. S2), suggesting that A20 is likely to stabilize NIK by interacting with the NIK ubiquitin-ligase complex composed of cIAP1/2, TRAF2, and TRAF3. To elucidate how A20 is involved in the attenuation of the interaction between NIK and cIAP1/2, TAP-A20-containing protein complexes were purified and components of the complex were analyzed. cIAP1 and TRAF2 but not cIAP2 and TRAF3 were co-precipitated with TAP-A20 (Fig. 3d). cIAP2 was not detected in the complex, most likely because of its low expression in HEK293T cells. However, the lack of TRAF3 in the A20-containing complex may suggest that association of A20 with either cIAP1/2 or TRAF2 results in the dissociation of the TRAF2/TRAF3 heterodimer, as confirmed and discussed below. To determine if a similar A20-containing complex forms under physiological conditions, endogenous A20 was immunoprecipitated in MDA-MB-231 cells before or after 3 h of LTα_1_β_2_ stimulation. Although A20 was co-precipitated with cIAP1/2 and TRAF2 in unstimulated cells, the amounts of cIAP1/2 and TRAF2 included in the A20-containing complex increased significantly with a concomitant increase in A20 upon stimulation induced by the canonical NF-κB pathway (Fig. 3e). By contrast, A20 was not associated with either TRAF3 or NIK (Fig. 3e), as demonstrated in the A20 overexpression experiment (Fig. 3d).

### The ZnF7 of A20 is crucial for cIAP1 binding and NIK stabilization

**Figure 4:**
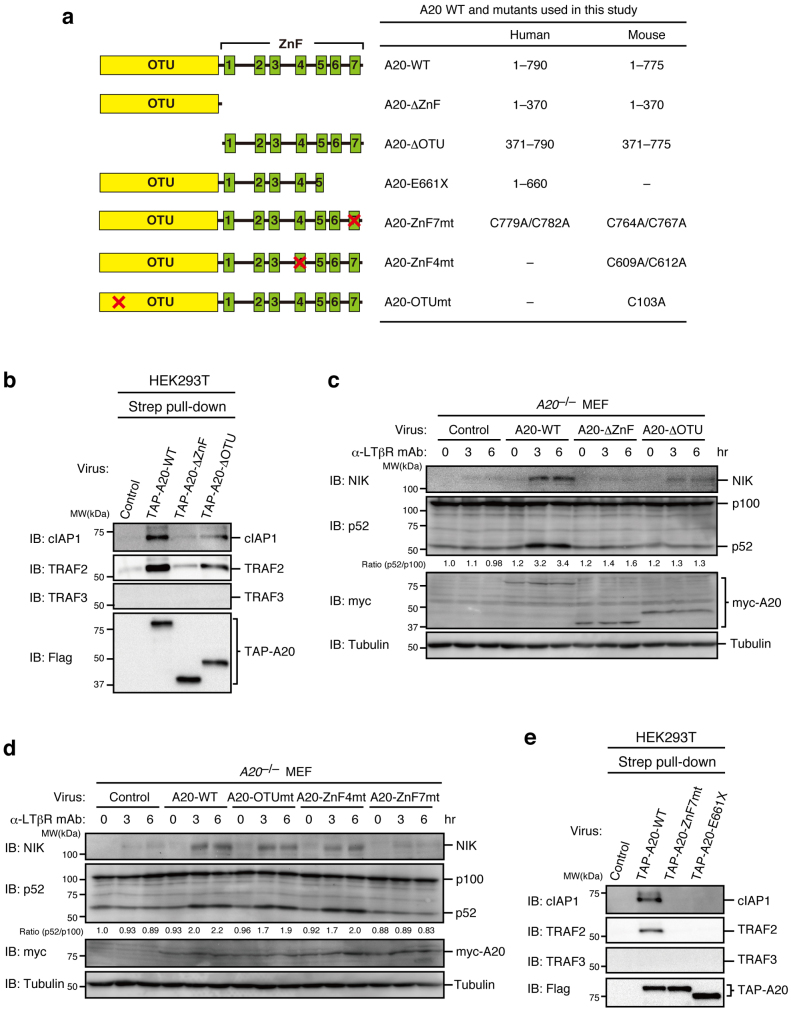
ZnF7 of A20 is crucial for the association of A20 with the cIAP1/TRAF2 complex and efficient activation of the non-canonical NF-κB pathway. (a) A schematic representation of various human and mouse A20 mutants used in this study. (b) and (e) The cell lysates prepared from HEK293T cells infected with control virus or virus expressing TAP-A20 or its mutant were subjected to affinity pull-down with Strep-Tactin Superflow beads. The precipitates were subjected to immunoblotting. (c) and (d) *A20*^−/−^ MEFs infected with control virus or virus expressing mouse wild type A20 or its mutant were stimulated with an agonistic anti-LTβR mAb (1 μg/ml) for the indicated times. Cell lysates were prepared and subjected to immunoblotting with the indicated antibodies. Quantitative ratios of p52 to p100 were calculated based on the data and are shown as ratios relative to those of the unstimulated control virus-infected cells. The depicted results are representative of three independent experiments.

Because A20 consists of an N-terminal OTU domain with deubiquitinase activity and a C-terminal ZnF region with E3 ligase activity^18^, we next determined which domain binds to the cIAP/TRAF2 complex. An A20-ΔOTU mutant lacking the OTU domain or an A20-ΔZnF mutant lacking all seven ZnFs (Fig. 4a) was expressed in HEK293T cells as a TAP-tagged protein, and affinity pull-down assays were performed. A20-ΔOTU but not A20-ΔZnF significantly bound to the cIAP1/TRAF2 complex (Fig. 4b). However, when either A20-ΔOTU or A20-ΔZnF was expressed in *A20*^−/−^ MEFs, neither mutant could compensate for the impaired activation of the non-canonical pathway in *A20*^−/−^ MEFs (Fig. 4c), indicating that the ZnF domain can associate with the cIAP1/TRAF2 complex but that the OTU domain is additionally required for activation of the non-canonical pathway. To further explore the role of A20 in the non-canonical pathway, we next addressed whether the catalytic activities of A20 are required for activation of the non-canonical pathway. An OTU domain mutant (A20-OTUmt) that lacks deubiquitinase activity because the catalytic Cys-103 was substituted with Ala and the fourth ZnF mutant (A20-ZnF4mt), which lacks E3 ligase activity because its conserved Cys residues (Cys-609, Cys-612) are substituted with Ala (Fig. 4a), were stably expressed in *A20*^−/−^ MEFs. LTβR stimulation-dependent NIK accumulation and p100 processing were similarly induced in cells expressing these mutants and A20-WT (Fig. 4d), indicating that neither the deubiquitinase nor the E3 ligase activity is required for the LTβR-induced activation of the non-canonical pathway. Because a recent study revealed that the seventh ZnF (ZnF7) domain of A20 is crucial for the binding of a K63 or linear polyubiquitin chain, thereby suppressing the canonical NF-κB pathway^20^,^21^,^22^, we analyzed *A20*^−/−^ MEFs expressing the ZnF7 mutant (A20-ZnF7mt) in which the conserved Cys residues (Cys-764, Cys-767) of ZnF7 are substituted with Ala (Fig. 4a). Expression of A20-ZnF7mt did not restore the non-canonical pathway in *A20*^−/−^ MEFs (Fig. 4d). In addition, the A20-ZnF7mt and the A20-E661X, in which the nonsense mutation reported in diffuse large B-cell lymphoma results in the generation of a C-terminally truncated protein at R660 (Ref. 23) (Fig. 4a), barely associated with the cIAP1/TRAF2 complex (Fig. 4e). These results indicate that the ZnF7 is essential for the association of A20 with the cIAP1/TRAF2 complex and the efficient activation of the non-canonical pathway.

### Direct binding of A20 and cIAP1/2 results in the dissociation of the TRAF2/TRAF3 heterodimer

**Figure 5:**
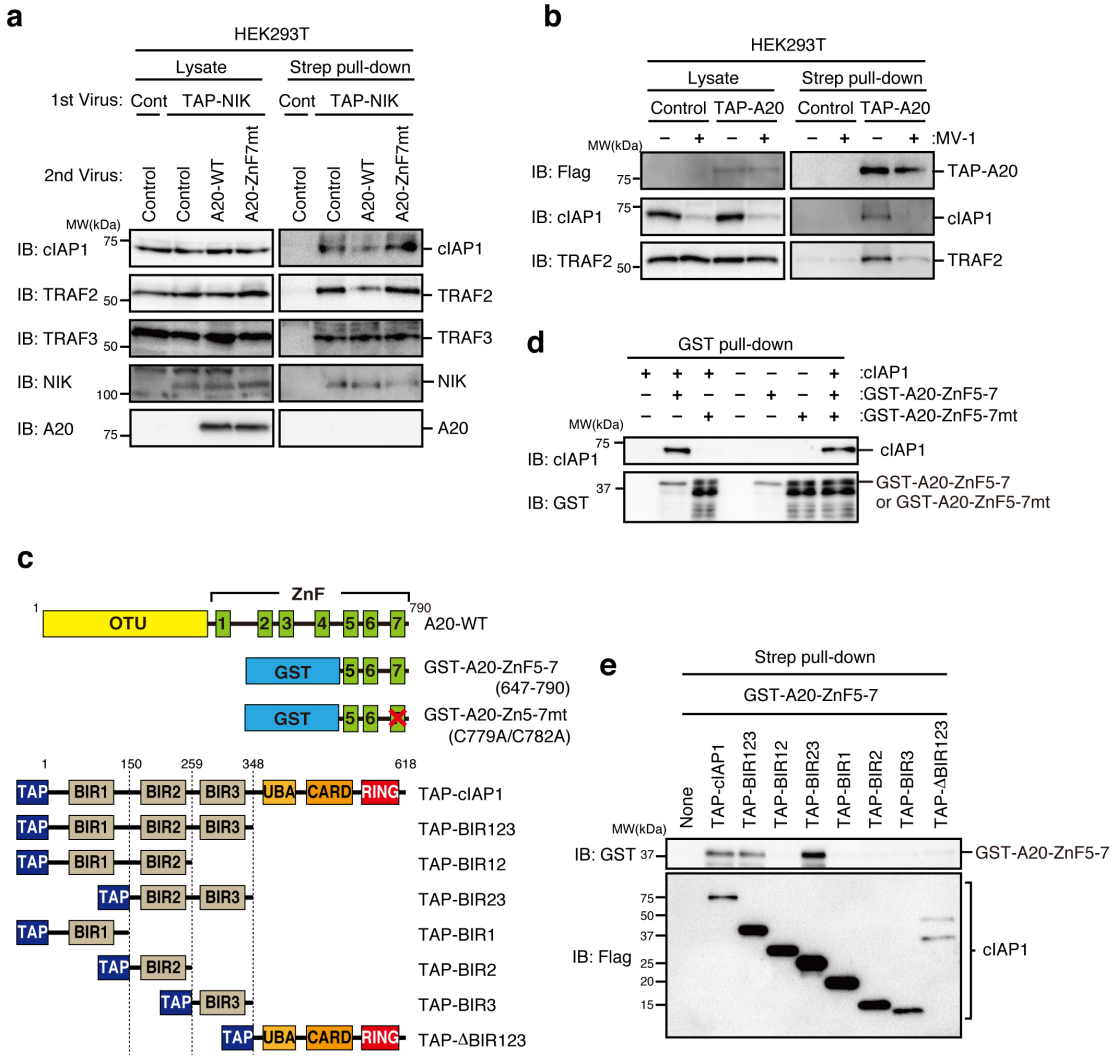
Direct binding of A20 to cIAP1 results in the dissociation of the TRAF2/TRAF3 interaction. (a) HEK293T cells were infected with either control virus or virus expressing TAP-NIK. The TAP-NIK expressing cells were further infected with control virus or virus expressing human wild type A20 or its mutant. Cell lysates were prepared and subjected to affinity pull-down with Strep-Tactin Superflow beads. The lysates and precipitates were subjected to immunoblotting. (b) HEK293T cells were infected with either control virus or virus expressing TAP-A20. The cells were untreated or treated with MV-1, a SMAC mimetic (5 μM), for 5 hr. The cell lysates were prepared and subjected to immunoblotting. (c) A schematic representation of various mutants of GST-human A20 and TAP-human cIAP1. (d) Various combinations of recombinant GST-A20-ZnF5-7, GST-A20-ZnF5-7mt, and cIAP proteins were incubated for 3 hr, and the reaction mixtures were subjected to GST pull-down assay. The precipitates were analyzed by immunoblotting with the indicated antibodies. (e) TAP-tagged cIAP1 or its mutant was transiently expressed in HEK293T cells and precipitated with Strep-Tactin Superflow beads. The precipitated beads were suspended and incubated with GST-A20-ZnF5-7 protein for 3 hr. The beads were then washed and subjected to immunoblotting. The depicted results are representative of three independent experiments. See also Supplementary Fig. S3.

Although several models for the stimulation-dependent stabilization of NIK have been proposed^15^,^16^,^24^,^25^, the critical event common to these models is the attenuation of the cIAP1/2-catalyzed K48 polyubiquitination of NIK resulting from the dissociation of the c-IAP1/2/TRAF2/TRAF3/NIK complex. Therefore, we next addressed whether A20 expression has any effect on complex formation because A20 overexpression triggers activation of the non-canonical pathway and concomitantly induces the association of A20 with the cIAP1/TRAF2 complex (Fig. 3a and d). N-terminally TAP-tagged NIK, alone or together with either Myc-A20-WT or Myc-A20-ZnF7mt, was stably expressed in HEK293T cells, and the NIK-associated proteins were analyzed by pull-down of TAP-NIK. TAP-NIK was associated with cIAP1, TRAF2, and TRAF3 in parental cells. The association of NIK with cIAP1 and TRAF2 but not with TRAF3 was significantly reduced by the over-expression of A20-WT but not A20-ZnF7mt (Fig. 5a). These results strongly suggest that the binding of A20 to cIAP1 through ZnF7 of A20 resulted in the dissociation of the complex by reducing the interaction between TRAF2 and TRAF3, thereby suppressing polyubiquitination and inducing the accumulation of NIK.

To further understand the mechanism of A20-mediated activation of the non-canonical pathway, the interaction between A20 and the cIAP1/TRAF2 complex was analyzed more precisely. We first attempted to determine which component of the cIAP1/TRAF2 complex binds A20. MV-1, a second mitochondria-derived activator of caspase (SMAC) mimetic that induces the depletion of cIAP1 by activating its auto-ubiquitination^26^, significantly inhibited the interaction of A20 with TRAF2 (Fig. 5b). Given that cIAP1 directly binds TRAF2 (Ref. 27), this result strongly suggests that A20 indirectly associates with TRAF2 via cIAP1. Because ZnF7 of A20 has been shown to play a critical role in the binding of A20 to K63 or linear polyubiquitin chains, we next addressed whether the binding between A20 and cIAP is direct or requires ubiquitin. Glutathione-S-transferase (GST) was fused to the fragment of A20 (aa 647–790) encompassing the ZnF5 to the ZnF7 (GST-A20-ZnF5-7), to the same fragment carrying the C779A/C782A mutation to inactivate the ZnF7 (GST-A20-ZnF5-7mt) (Fig. 5c), and to the whole cIAP1 (GST-cIAP) and expressed in *E. coli* (Supplementary Fig. S3). Because *E. coli* does not produce ubiquitin, recombinant proteins prepared from *E. coli* are not ubiquitinated or co-purified with ubiquitin. Bead-bound GST-cIAP1 was then cleaved with 3C protease to release cIAP1 (Supplementary Fig. S3), which was then mixed with GST-A20-ZnF5-7 or its mutant and followed by a GST pull-down assay. cIAP1 directly bound GST-A20-ZnF5-7 but not GST-A20-ZnF5-7mt. cIAP was also pulled down when GST-A20-ZnF5-7 was mixed with GST-A20-ZnF5-7mt, indicating that the degradation products of GST-A20-ZnF5-7mt did not inhibit the interaction between cIAP1 and A20-ZnF5-7 (Fig. 5d). These results indicate that A20 directly binds to cIAP1/2 in the absence of ubiquitin. To determine the domain of cIAP1 to which A20-ZnF5-7 binds, TAP-tagged cIAP1 and its deletion mutants were expressed in HEK293T cells and partially purified with Strep-Tactin beads (Fig. 5c). These beads were then incubated with GST-A20-ZnF5-7 proteins, and pull-down assays were performed to assess the association between the cIAP1 deletion mutants and GST-A20-ZnF5-7. The baculovirus IAP repeat 2 (BIR2) and BIR3 domains are each required for the binding of cIAP1 to A20, and together, they are sufficient for binding (Fig. 5e). Given that TRAF2 binds the BIR1 domain^27^, these results are consistent with the formation of the A20/cIAP1/TRAF2 complex before and after LTβR stimulation (Fig. 3e).

### K63 polyubiquitin, but not linear polyubiquitin, and cIAP1 competitively bind to A20

**Figure 6:**
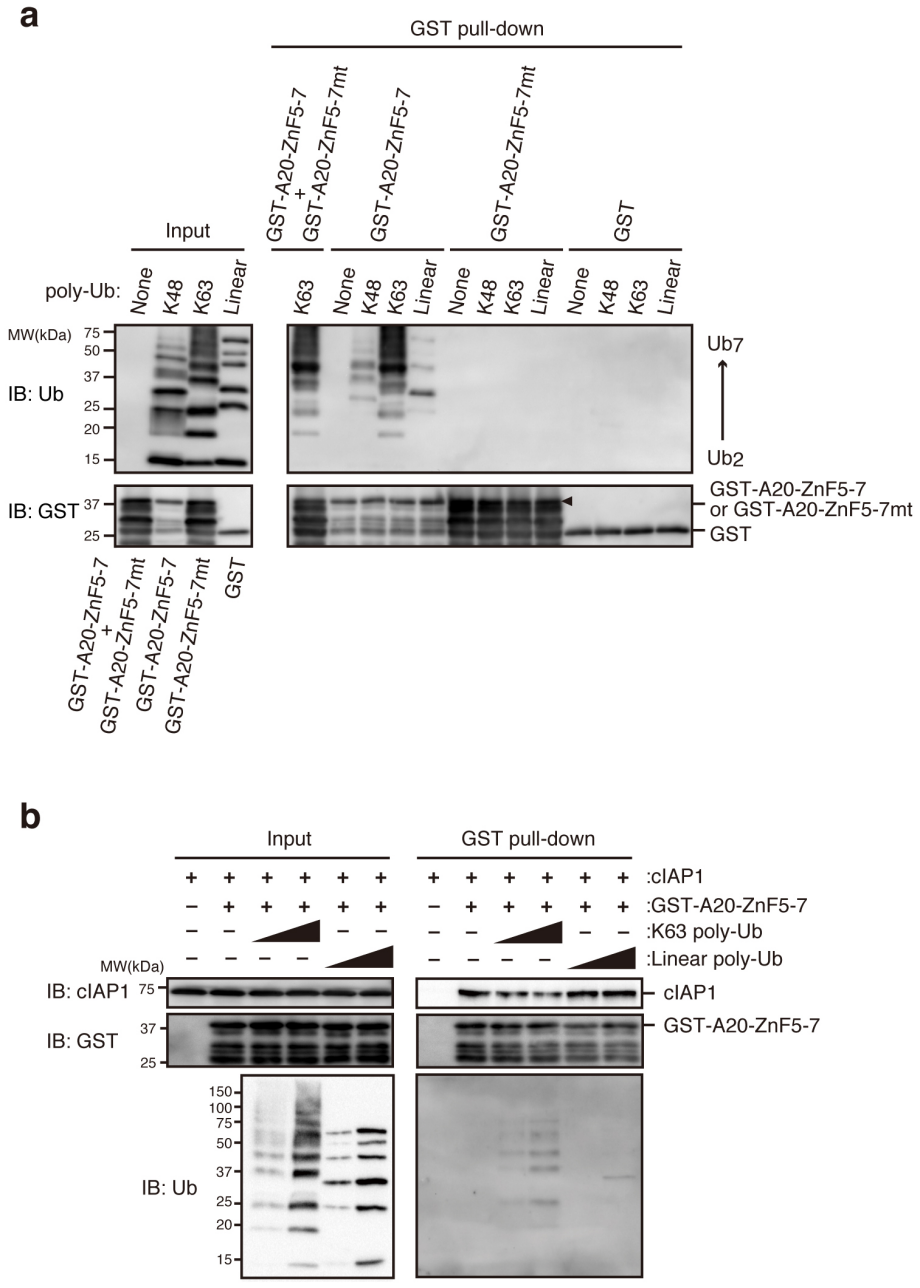
K63 polyubiquitin, but not linear polyubiquitin, and cIAP1 competitively bind to ZnF7 of A20. (a) GST-A20-ZnF5-7, GST-A20-ZnF5-7mt, GST, or the mixture of GST-A20-ZnF5-7 and its mutant was incubated with the indicated polyubiquitin chains for 3 hr and subjected to a GST pull-down assay. Aliquots of the reactions (input) and the precipitates were subjected to immunoblotting with the indicated antibodies. The arrowhead indicates full-length proteins of GST-A20-ZnF5-7 and its mutant. Loading amounts were adjusted to achieve roughly equal amounts of full-length bands of GST and GST fusion proteins. See Supplementary Fig. S3b and S3c. (b) Recombinant cIAP1 protein (0.5 μg) was incubated with GST-A20-ZnF5-7 (0.5 μg) for 1 hr in the presence (0.5 or 3 μg) or absence of the indicated polyubiquitin chains. After 3 hr, the samples were subjected to a GST pull-down assay, and aliquots of the reactions (input) and the precipitates were analyzed by immunoblotting with the indicated antibodies. The depicted results are representative of three independent experiments. See also Supplementary Fig. S3.

ZnF7 has been reported to bind to either K63 polyubiquitin or linear polyubiquitin^20^,^21^,^22^. Given that these polyubiquitin chains are generated during the activation of the canonical pathway^2^, one may speculate that these polyubiquitin chains modulate the activation of the non-canonical pathway by competing against cIAP1/2 upon binding to A20. We first assessed which of the two chain types could bind efficiently to ZnF7 by performing the GST pull-down assay after incubation of GST-A20-ZnF5-7 with recombinant polyubiquitin chains. In contrast to the previous report^21^,^22^, K63 polyubiquitin binds to A20 more efficiently than the linear polyubiquitin chain (Fig. 6a). Moreover, the addition of recombinant K63 polyubiquitin chains but not linear polyubiquitin chains inhibited the interaction between ZnF7 and cIAP1 (Fig. 6b), indicating that cIAP and K63 polyubiquitin chains bind to ZnF7 competitively. These results suggest a novel regulatory role of the K63 polyubiquitin chain in the non-canonical NF-κB pathway.

## Discussion

**Figure 7:**
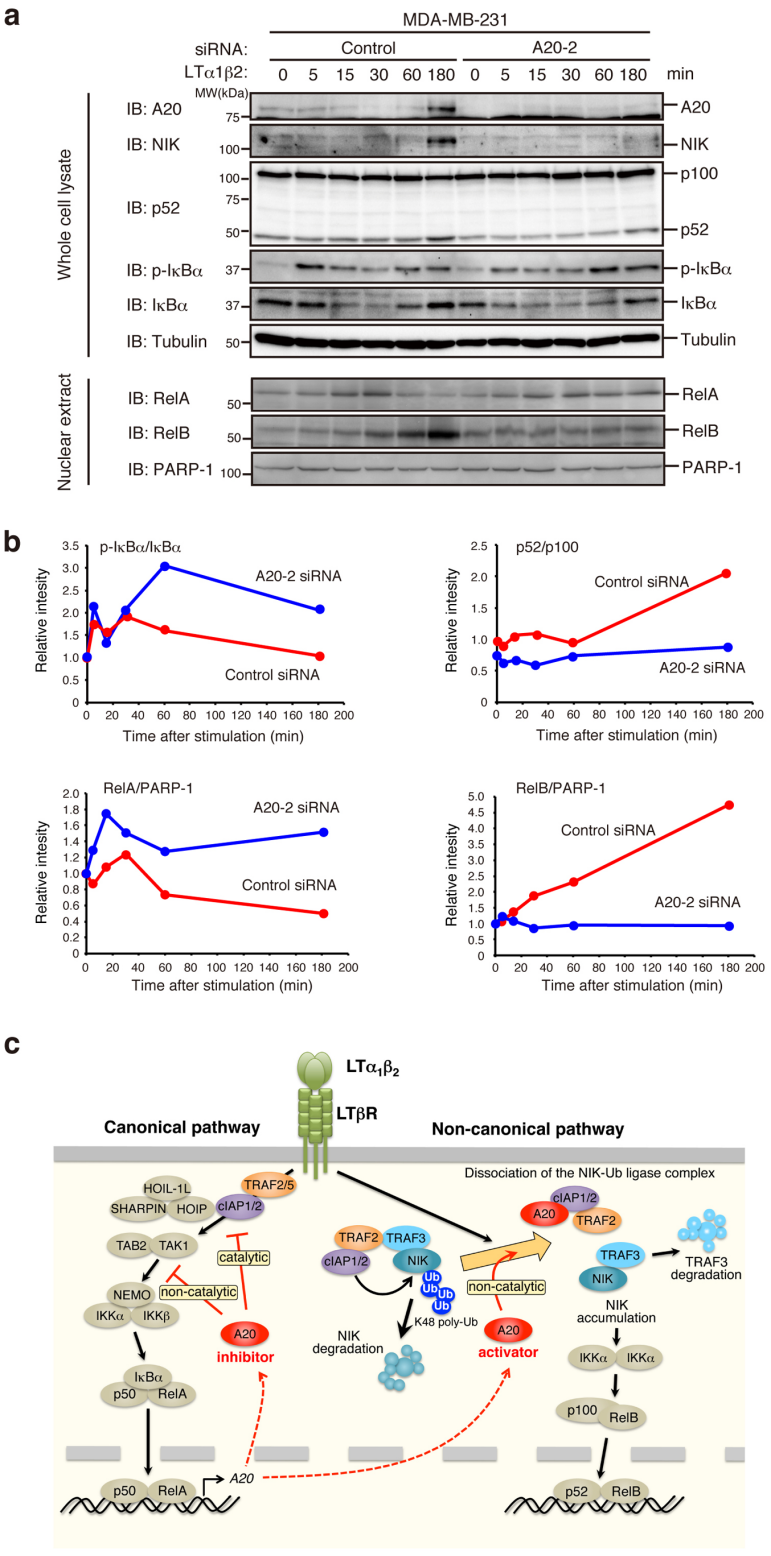
A20 promotes switching from the canonical to the non-canonical NF-κB pathway upon LTβR stimulation. (a) MDA-MB-231 cells were transfected with either control siRNA or A20 siRNA (A20-2). After 72 hr, the cells were stimulated with recombinant LTα_1_β_2_ (500 ng/ml) for the indicated time. Whole cell lysates and nuclear extracts were then prepared and subjected to immunoblotting with the indicated antibodies. (b) Quantitative ratios of phospho-IκBα to total IκBα (upper left), p52 to p100 (upper right), RelA to PARP-1 (lower left), and RelB to PARP-1 (lower right) were calculated based on the data shown in (a) and are shown as ratios relative to those of the unstimulated control siRNA-transfected cells. The depicted results are representative of three independent experiments. (c) A schematic model illustrating the A20-mediated switching from the canonical to the non-canonical NF-κB pathway (see the text for the details).

Although only a specific subset of TNFRSF activates the non-canonical NF-κB pathway, this pathway plays critical and unique roles in establishing the immune system. In general, the canonical pathway rapidly activates the p50/RelA heterodimer, whereas the non-canonical pathway slowly activates the p52/RelB heterodimer afterwards (Fig. 7a and b). Due to these differences, the two pathways have distinct target gene specificities and gene expression kinetics, which are crucial for the proper regulation of inflammation and immune responses^28^. Therefore, elucidation of the molecular mechanism by which the non-canonical pathway becomes activated is of particular interest. A critical event that triggers the non-canonical pathway is the attenuation of NIK degradation, resulting from K48 polyubiquitination of NIK catalyzed by the NIK ubiquitin ligase complex, which includes TRAF2, TRAF3, and cIAP1/2. Genetic deficiency in any component of the ligase complex leads to the stabilization of NIK and activation of the non-canonical pathway^29^,^30^,^31^,^32^. In this paper, we clearly demonstrate that A20-deficiency significantly abrogates NIK accumulation and p100 processing to p52 that were induced by stimulation of LTβR, Fn14, and RANK. Furthermore, shifting from the canonical to the non-canonical pathway in LTβR signaling is not properly executed in A20 knockdown cells (Fig. 7a and b). These results strongly suggest that A20 is involved in the molecular switch that promotes the transition from canonical to non-canonical activation (Fig. 7c). Therefore, A20 is a double-edged sword: it is a positive regulator of the non-canonical pathway due to its involvement in the attenuation of NIK degradation as well as a negative regulator of the canonical pathway as identified previously.

The deubiquitinase and ubiquitin ligase activities of A20 are not required for the A20-mediated enhancement of non-canonical NF-κB activation, but ZnF7 is essential. In this sense, our observation is similar to the previously reported noncatalytic mechanisms of IKK inhibition by A20 in the canonical pathway^20^,^21^,^22^. However, one of the critical findings in this study is that ZnF7 binds cIAP1/2 in addition to its previously recognized binding to either K63 or linear polyubiquitin chains. Interestingly, the binding of cIAP1/2 to A20 was competitively blocked by the K63 polyubiquitin chain, suggesting that unanchored or substrate-conjugated K63 polyubiquitin chains generated during the activation of the canonical pathway^5^ may block the interaction of A20 with cIAP1/2, thereby blocking early activation of the non-canonical pathway.

How is A20 involved in the inactivation of the NIK ubiquitin ligase complex? Our results suggest that interaction of A20 with cIAP1/2 may lead to the dissociation of the TRAF2/TRAF3 interaction within the complex. Based on previous literature, the BIR1 domain of cIAP1/2 binds to the coiled-coil domain of TRAF2 (Ref. 27), which partially overlaps the TRAF3 binding domain in TRAF2 (Ref. 33). Moreover, our data indicate that ZnF7 of A20 binds to the BIR2 and BIR3 domains, which are adjacent to the BIR1 domain. Therefore, the interaction of A20 with cIAP1/2 may inhibit the TRAF2/TRAF3 interaction through structural changes in TRAF2 induced by the indirect interaction of A20 with TRAF2 via the BIR domains of cIAP1/2 or by the direct interaction between A20 and TRAF2, as suggested by the yeast two-hybrid experiment^34^.

Overexpression of A20 induced by transient transfection of the TAP-A20 expression vector into HEK293T cells was able to induce p100 processing and NIK accumulation without stimulation. Moreover, several hours after LTβR stimulation, A20 expression was significantly augmented with concomitant accumulation of NIK. These results might suggest that the accumulation of A20 upon stimulation by LTβR could be sufficient to trigger the non-canonical pathway, thereby resulting in slower kinetics of the non-canonical pathway in comparison with that of the canonical pathway. However, the following observations indicate that this is not the case: first, TNFα or IL-1 stimulation does not activate the non-canonical pathway, while both can induce A20 efficiently^2^,^35^; second, the non-canonical pathway was activated with slow kinetics similar to that observed in wild-type MEFs and even in *A20*^−/−^ MEFs complemented with A20 in which a sufficient amount of A20 is constitutively expressed irrespective of LTβR stimulation. These results indicate that the A20 accumulation in response to physiological stimulation is not sufficient and that other cooperative signal(s) triggered by the specific subset of TNFRSF is/are required for non-canonical activation.

It has been reported that stimulation-induced TRAF3 degradation is involved in NIK accumulation^15^,^16^. In addition, both the activation of the non-canonical LTβR pathway and TRAF3 degradation require receptor internalization^24^. However, we demonstrated that overexpression of A20 does not induce TRAF3 degradation but does induce non-canonical activation. Moreover, stimulation-induced TRAF3 degradation was not affected in the absence of A20. Therefore, TRAF3 degradation and receptor internalization are independent of A20 and may not be required for non-canonical activation at least in the cells used in this paper. Co-operation of A20-mediated destabilization of the TRAF2/TRAF3 complex and the A20-independent but receptor stimulation-dependent event(s) that is/are somehow associated with TRAF3 degradation could be essential for non-canonical activation in response to physiological stimulation. It has been reported that LTβR and NIK bind to TRAF3 at the same site and that LTβR stimulation results in the recruitment of TRAF3 to the oligomerized LTβR, causing the competitive displacement of NIK from TRAF3 and preventing NIK degradation by cIAP1/2 (Ref. 25). This receptor-dependent event is likely to cooperate with A20.

Gene-knockout experiments revealed an essential role for the non-canonical LTβR pathway in lymph node development^36^,^37^. However, although A20-deficient mice were shown to have multiorgan inflammation, severe cachexia, and premature lethality, defects in lymph nodes have not been reported^38^. This result may be a consequence of mild lymph-node defects resulting from the residual activation of the non-canonical LTβR pathway in the absence of A20. Therefore, precise analysis of the conditional *A20*^−/−^ mice is required.

Based on the analysis of the *A20* gene in various tumors, it has been proposed that elevated activation of NF-κB by inactivation of A20 could be involved in cancer development^23^,^39^. By contrast, our results strongly suggest that excess expression of A20 could result in enhanced activation of the non-canonical NF-κB pathway, the excess activation of which is tightly linked to tumor formation^4^. We quantified the level of NF-κB activation in various breast cancer cell lines and reported that they were significantly high in triple-negative breast cancer, the most malignant type of breast cancer^40^. Based on our experimental data from DNA microarrays measuring non-canonical activation, seven of eight cell lines with the highest NF-κB activation show both constitutive activation of the non-canonical pathway and high expression of A20 (Supplementary Fig. S4). Therefore, activation or enhanced expression of A20, as well as its inactivation, is likely to cause tumor development *in vivo*. Although further experiments are required to support this hypothesis, our elucidation of the critical role of A20 may contribute to the identification of novel regulation of the NF-κB pathway and may facilitate the development of therapeutic strategies for various diseases caused by the dysregulation of NF-κB.

## Methods

### Reagents and plasmids

Recombinant human LTα1β2 and TWEAK were purchased from R&D Systems. RANKL was purchased from Wako. Anti-mouse LTβR antibody was purchased from Enzo Life Sciences. MV1 was kindly provided by Y. Demizu (National Institute of Health Sciences, Japan). Human cDNAs encoding A20 (wild type and mutants), cIAP1 (wild type and mutants), CD40, and NIK and mouse cDNAs encoding A20 (wild type and mutants) and RANK were generated by PCR and inserted into the retroviral vector pMXs, which was obtained from T. Kitamura (University of Tokyo, Japan). A TAP (Flag-Strep-Strep) tag or myc tag was added at the N-terminus of the genes as indicated in the text. Control siRNA and siRNA for A20 (#1, 5′-CCC AGC UUU CUC UCA UGG AUG UAA A-3′; #2, 5′-GCC UGA AAU CCG AGC UGU UCC ACU U-3′) were purchased from Life Technologies. An siRNA-resistant A20 cDNA that carries seven silent mutations was generated by PCR to change the sequence from 5′-GCC TGA AAT CCG AGC TGT TCC ACT T-3′ to 5′-GCC aGA gAT aCG tGC aGT aCC tCT T-3′.

### Cell culture and transfection

*A20*^−/−^ and control MEFs were kindly provided by Dr. A. Ma (University of California at San Francisco, USA). The cell culture conditions were described previously^40^,^41^. siRNA (33 nM) was transfected into cells with Lipofectamine RNAiMAX (Life Technologies) according to the manufacturer's instructions. For retrovirus-mediated gene transfer, the packaging Plat-A and Plat-E cells were transfected with pMXs plasmids, and virus stocks were prepared by collecting the culture medium. Cells were infected with the virus stock in the presence of 10 μg/ml polybrene (Sigma-Aldrich), followed by selection with puromycin, and the resultant cell pools were used. For co-expression experiments, the obtained cell pools were again infected with virus stocks, followed by selection with blasticidin.

### Quantitative real-time reverse transcriptase PCR analysis

Total RNA isolation and reverse transcription were described previously^40^. The following primers were used: human MAP3K14 forward/reverse, 5′-CGG AAA GTG GGA GAT CCT GAA-3′/5′-GGG CGA TGA TAG AGA TGG CAG-3′; human GAPDH forward/reverse 5′-AAG GTG AAG GTC GGA GTC AAC-3′/5′-GGG GTC ATT GAT GGC AAC AAT A-3′; mouse Map3k14 forward/reverse, 5′-TGT GGG AAG TGG GAG ATC CTA-3′/5′-GGC TGA ACT CTT GGC TAT TCT CA-3′; mouse β-actin forward/reverse, 5′-GGC TGT ATT CCC CTC CAT CG-3′/5′-CCA GTT GGT AAC AAT GCC ATG T-3′. After initial denaturation at 95°C for 1 min, PCR was performed for 40 cycles (15 s at 95°C and 45 s at 60°C) using the Thunderbird SYBR Green Polymerase Kit (Toyobo) and ABI prism 7300 Real Time PCR System (Life Technologies).

### Immunoblotting and cell fractionation

Immunoblotting and cell fractionation were described previously^40^. The antibodies used for immunoblot analysis were as follows: anti-myc, anti-TRAF3, anti-IκBα, anti-RelA, and anti-RelB (Santa Cruz Biotechnology); anti-NIK, anti-cIAP2, anti-TRAF2, anti-p100, anti-A20, anti-phospho-IκBα, anti-PARP1, and anti-phospho-IKK (Cell Signaling Technology); anti-tubulin and anti-p100 (Millipore); anti-cIAP1 (Enzo Lifesciences); anti-A20 (eBiosciences); anti-FLAG (Sigma-Aldrich); and horseradish peroxidase-conjugated secondary antibodies (GE Healthcare). For quantitation, bands were analyzed with Image Lab 3.0 software (Bio-Rad).

### GST pull-down assay

Human cDNAs encoding A20 ZnF5-7, ZnF5-7mt, and cIAP1 were generated by PCR and inserted into pGEX-6P-1. GST and GST-fused proteins were expressed in *E. coli* DH5α and purified using Glutathione Sepharose 4B (GE Healthcare). The GST tag was removed from cIAP1 by Turbo3C protease (Wako). GST-ZnF5-7 proteins were dialyzed with PBS. K48-linked and K63-linked polyubiquitin chains (Ub_2-7_) were purchased from Boston Biochem. Linear polyubiquitin chains (Ub_2-7_) were purchased from Enzo Lifesciences. A quantity of 2 μg of polyubiquitin chains or 0.5 μg of cIAP1 proteins was incubated with 0.5 μg of GST or GST-ZnF5-7 and 10 μl of Glutathione Sepharose 4B in TNE buffer [20 mM Tris–HCl (pH 7.5), 130 mM NaCl, 1% Triton X-100, 1 mM EDTA, 2 mM Na_3_VO_4_, 10 mM NaF] containing 0.5 mM DTT and 0.1 mg/ml BSA for 3 hr. After incubation, the beads were washed three times with TNE buffer and then suspended in sample buffer. For competition experiments, 0.5 μg of cIAP1 protein was incubated with 0.5 μg of GST-ZnF5-7 and 10 μl of Glutathione Sepharose 4B in TNE buffer with 0.5 mM DTT and 0.1 mg/ml BSA for 1 hr, and then 0.5 or 3 μg of K63-linked polyubiquitin chains were added to the mixture. After a 3-hr incubation, the beads were washed three times with TNE buffer and then suspended in sample buffer.

### Immunoprecipitation and Strep-Tactin pull-down

Cells were lysed in TNE buffer and centrifuged to remove cellular debris. The resulting supernatant was mixed with 1 μg of the appropriate antibody together with 10 μl of protein G Sepharose beads (GE Healthcare) or 10 μl of Strep-Tactin Superflow beads (IBA, USA). After overnight incubation, the beads were washed three times with TNE buffer and then suspended in sample buffer. To assess the binding of TAP-cIAP1 and GST-ZnF5-7, precipitated samples were incubated with 0.5 μg of GST-ZnF5-7 in TNE buffer with 0.5 mM DTT and 0.1 mg/ml BSA for 3 hr, washed three times with TNE buffer, and suspended in sample buffer.

### Tandem affinity purification and mass spectrometry

HEK293T cells expressing TAP-A20 were lysed in TNE buffer with protease inhibitor cocktail (Roche), and cell debris was removed by centrifugation at 10,000 × *g* for 15 min to prepare the cell lysate. Protein complexes containing A20 were precipitated with Strep-Tactin Superflow beads and eluted with desthiobiotin. The eluates were treated with anti-Flag M2 beads, and the protein complexes were eluted with Flag peptides. The eluted proteins were digested with trypsin and loaded onto an automated nanoflow liquid chromatography system (Dina) coupled to a linear ion trap-orbitrap mass spectrometer (LTQ-Orbitrap Velos, Thermo Fisher Scientific). The tandem mass spectrometry signals were processed against human protein sequences in the NCBI RefSeq database using the Mascot algorithm (Matrix Science).

### Statistical analysis

Statistically significant differences between the mean values were determined using Student's *t*-test. Data are presented as the means ± SD.

## Author Contributions

N.Y. performed most of the experiments. M.O. and H.K.-H. performed the proteomic analysis. N.Y. and J.I. designed the study, analyzed the data, and wrote the paper.

## Supplementary Material

Supplementary Information Supplementary Information
