# Supplementary material for: Involvement of A20 in the molecular switch that activates the non-canonical NF-кB pathway
Source: Sci Rep. 2013 Sep 6;3:2568. doi: 10.1038/srep02568 (PMC3764444; doi:10.1038/srep02568)
Supplement: Supplementary Information [file srep02568-s1.pdf]

Supplementary information

Involvement of A20 in the molecular switch that activates the non-canonical NF-κB pathway

Noritaka Yamaguchi, Masaaki Oyama, Hiroko Kozuka-Hata, and Jun-ichiro Inoue

Supplementary Figures S1-S4
Supplementary References

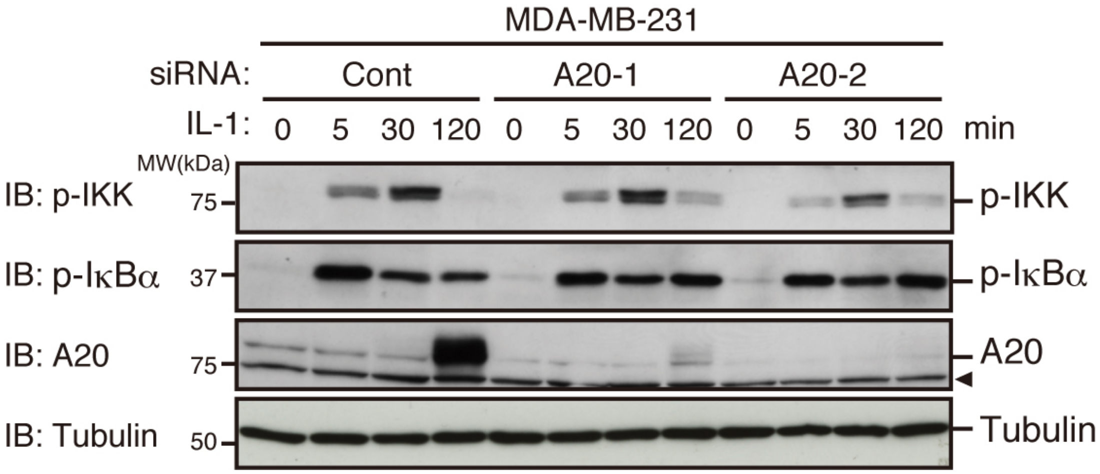

Supplementary Figure S1. A20 knockdown resulted in the prolonged activation of the canonical NF-κB pathway by IL-1 stimulation in the late phase.

MDA-MB-231 cells were transfected with the indicated siRNAs. After 72 hr, the cells were untreated or treated with recombinant IL-1 (10 ng/ml) for the indicated time. Cell lysates were then prepared and subjected to immunoblotting with the indicated antibodies. An arrowhead denotes non-specific bands. The depicted results are representative of three independent experiments.

a

b

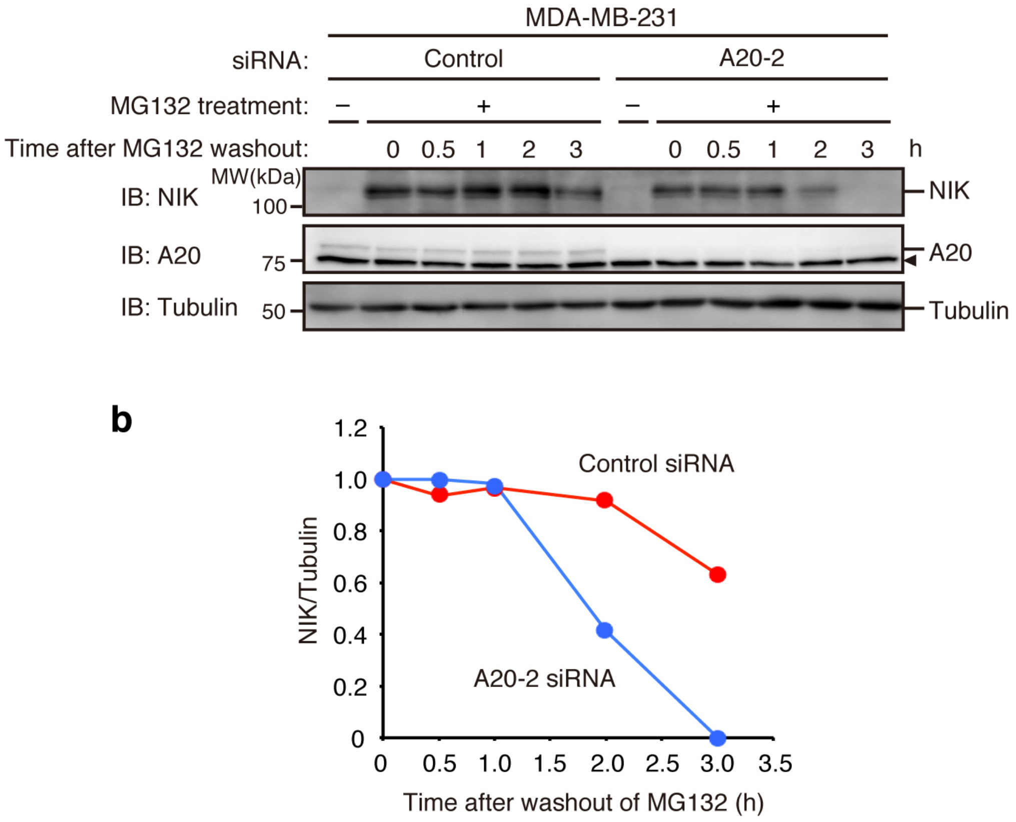

Supplementary Figure S2. A20 stabilizes NIK protein.

MDA-MB-231 cells were transfected with the indicated siRNAs. After 72 hr, the cells were untreated or treated with MG132 (10 μM) for 2 hr. Cells were then washed two times with MG132-free media and then further incubated in MG132-free media supplemented with cycloheximide (10 μg/ml) for the indicated times. Cell lysates were then prepared and subjected to immunoblotting with the indicated antibodies (a). An arrowhead denotes non-specific bands. Quantitative ratios of NIK to tubulin were calculated based on the data shown in (a) and are shown as ratios relative to those of the MG132-treated, unwashed cells (b). The depicted results are representative of three independent experiments.

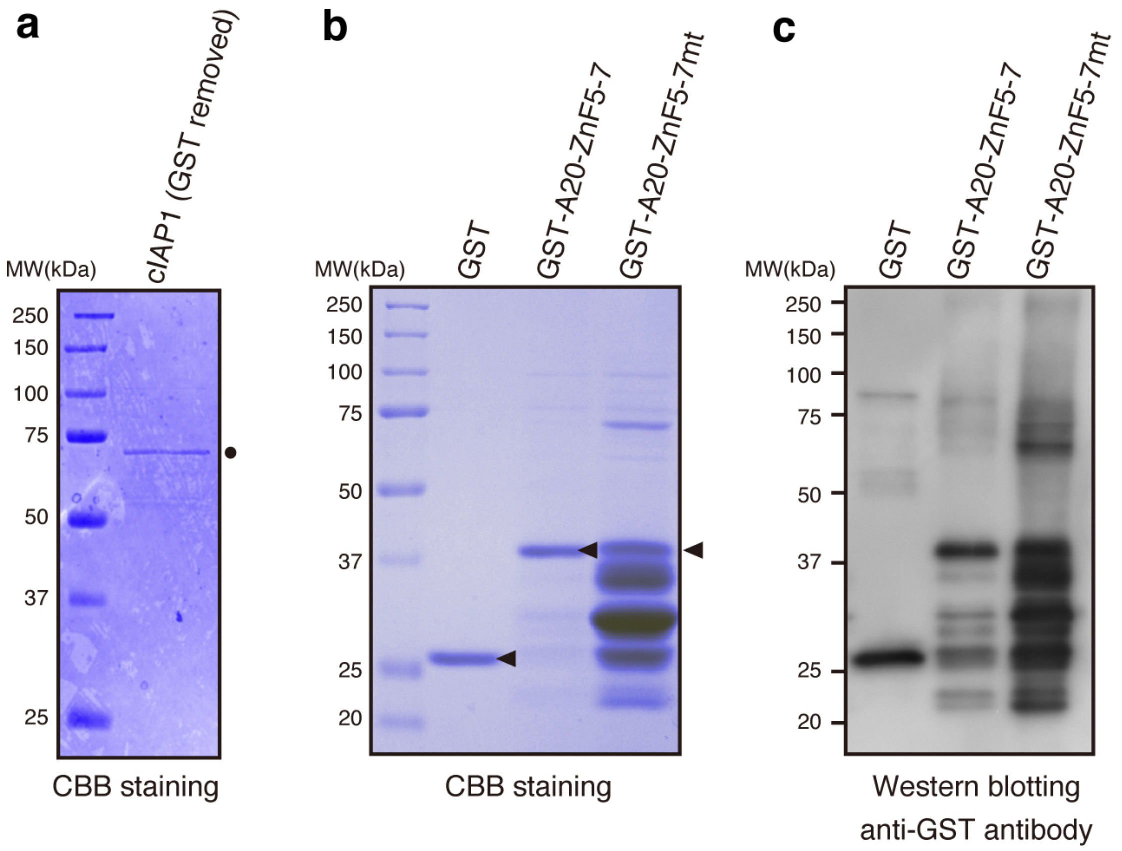

Supplementary Figure S3. Characterization of purified GST, GST-A20-ZnF5-7, GST-A20-ZnF5-7mt, and cIAP1 proteins.

GST, GST-A20-ZnF5-7, GST-A20-ZnF5-7mt, and GST-cIAP1 were expressed in *E. coli* and purified with Glutathione Sepharose beads. GST-cIAP1 proteins were digested with Turbo3C protease, and GST-free cIAP1 proteins were collected. GST and GST fusion proteins were eluted with glutathione and dialyzed against PBS. Aliquots of GST-removed cIAP1 (a), GST, GST-A20-ZnF5-7, and GST-A20-ZnF5-7mt (b) were separated on polyacrylamide/10% SDS gel and visualized by Coomassie brilliant blue R-250 (CBB) staining. A dot denotes GST-removed cIAP1 protein and arrowheads indicate full-length forms of GST and GST fusion proteins. Note that similar amounts of full-length proteins are applied on the SDS gel shown in (b). The same amounts of the GST proteins used in (b) were analyzed by immunoblotting with anti-GST antibody (c).

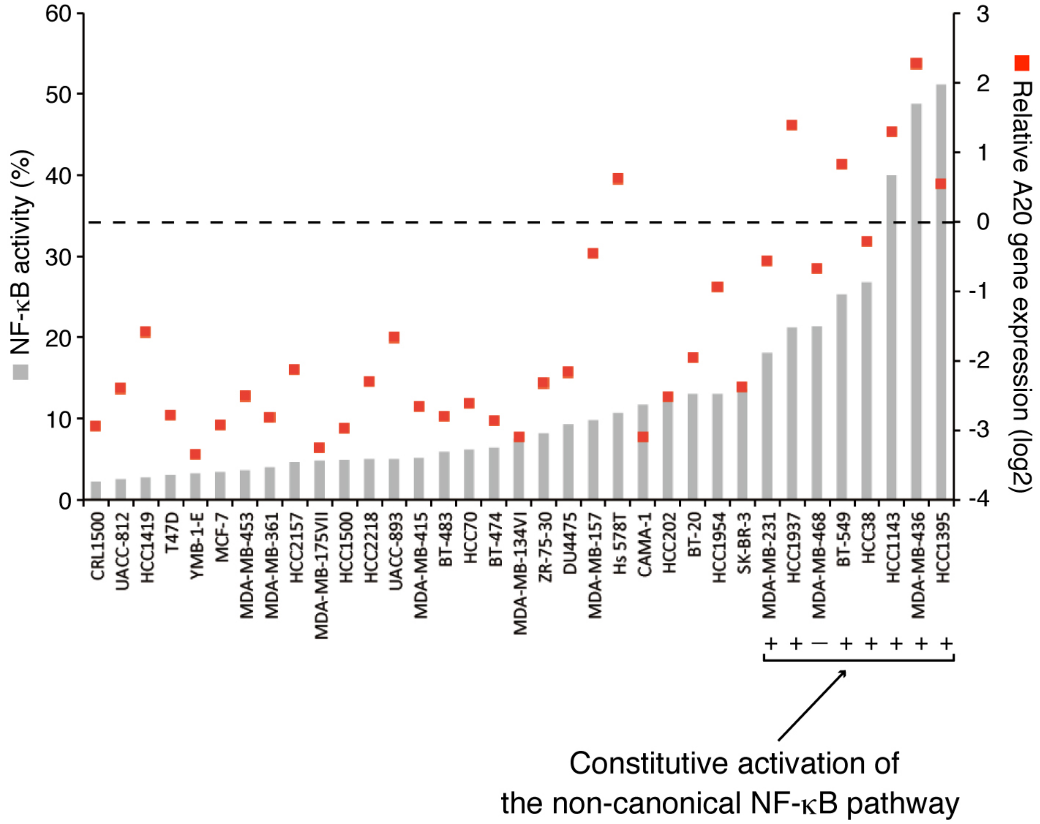

Supplementary Figure S4. Correlation between the levels of A20 mRNA expression and those of constitutive NF-κB activation in various human breast cancer cell lines. The levels of NF-κB activation measured by electrophoretic mobility shift assays (EMSAs) as previously described[1] are shown on the left y-axis; NF-κB activation in TNFα-stimulated Jurkat cells was set to 100. The relative expression levels of the A20 gene, which were estimated based on the previous microarray data[2], are shown on the right y-axis. Eight cell lines with high NF-κB activation were tested for the level of non-canonical activation based on the results of EMSA supershift experiments with antibodies against RelB or p52 (Ref. 1). Cells with (+) or without (–) constitutive activation of the non-canonical pathway are shown.

Supplementary References

1. Yamaguchi, N. *et al*. Constitutive activation of nuclear factor-κB is preferentially involved in the proliferation of basal-like subtype breast cancer cell lines. *Cancer Sci.* **100**, 1668-1674 (2009).
2. Ito, E. *et al*. Novel clusters of highly expressed genes accompany genomic amplification in breast cancers. *FEBS lett.* **581**, 3909-3914 (2007).
